# CTNNB1 and CDH1 Regulate Trophoblast Cell Adhesion and Junction Formation in Yak Placental Tissue at Different Gestational Stages

**DOI:** 10.3390/ani15060876

**Published:** 2025-03-19

**Authors:** Bohao Zhang, Chen Song, Bin Zhou, Junjun Zhang, Weitao Dong, Yong Zhang, Xingxu Zhao, Quanwei Zhang

**Affiliations:** 1 College of Veterinary Medicine, Gansu Agricultural University, Lanzhou 730070, China; zhangbhgs@163.com (B.Z.); scott1217163@163.com (C.S.); d.wt2008@163.com (W.D.); zhangy@gsau.edu.cn (Y.Z.); zhaoxx@gsau.edu.cn (X.Z.); 2 College of Life Science and Biotechnology, Gansu Agricultural University, Lanzhou 730070, China; zhoub@st.gsau.edu.cn (B.Z.); 107332202298@st.gsau.edu.cn (J.Z.); 3 Gansu Key Laboratory of Animal Generational Physiology and Reproductive Regulation, Lanzhou 730070, China

**Keywords:** yak, placenta, DIA proteomics, β-catenin (CTNNB1), E-cadherin (CDH1), bioinformatic analysis

## Abstract

The molecular mechanisms underlying trophoblast (TC) adhesion and junction formation, essential processes for mammal placental development and pregnancy maintenance, are unclear in yaks. Here, we investigated adhesion- and junction-related phenotypes in placental tissue and identified differentially expressed proteins (DEPs) related to TC adhesion and junction formation through data-independent acquisition proteomics analysis, then validated DEP functions and expression patterns through morphological and molecular biology techniques. Yak placental tissues at five months of gestation showed enhanced TC proliferation and differentiation compared to those at two months of gestation, and upregulated intercellular adhesion molecule-1 and occludin, highlighting the importance of cell adhesion and junction formation in TC differentiation and placental development. We identified 54 downregulated and 49 upregulated DEPs enriched in six Gene Ontology terms related to cell adhesion and junction formation, with β-catenin (CTNNB1) and E-cadherin (CDH1) playing pivotal roles. Bioinformatic analysis of Kyoto Encyclopedia of Genes pathways suggested that CTNNB1 and CDH1 are involved in cell adhesion and cancer, with implications for placental development. Immunohistochemistry and immunofluorescence staining revealed the strong expression of CTNNB1 and CDH1 in TC cytoplasm, particularly in trophoblast giant cells and uninucleate TCs at five months of gestation. qPCR and Western blot analyses revealed upregulated *CTNNB1* and *CDH1* mRNA and protein levels at five months of gestation, implicating their critical role in maintaining placental homeostasis and TC adhesion and junction formation. These findings elucidate the molecular mechanisms by which cell adhesion and junction formation regulate TC function and placental development during yak gestation.

## Simple Summary

As a major cell type in the placenta, trophoblasts (TCs) play a crucial role in supporting placental development and fetal growth. Investigating the molecular mechanisms underlying TC adhesion and junction formation during different gestational stages is essential for improving the reproductive health of yaks. In this study, candidate differentially expressed proteins associated with cell adhesion and junction formation were identified using data-independent acquisition proteomics analysis. Bioinformatic analysis of six Gene Ontology terms and two Kyoto Encyclopedia of Genes pathways revealed that β-catenin (CTNNB1) and E-cadherin (CDH1) are involved in cell adhesion and junction formation, influencing both placental development and the pregnancy process. High expression of CTNNB1 and CDH1 in the TCs of placental tissues at five months of gestation confirmed their critical role in maintaining pregnancy homeostasis. This study provides new insights into the molecular mechanisms underlying TC adhesion and junction formation during yak placental development.

## 1. Introduction

Yaks (*Bos grunniens*), which are distributed across the Tibetan Plateau and other high-altitude regions, are vital livestock that provide essential resources for local herders and have significant economic and ecological value [1,2]. However, reproductive diseases, such as miscarriage, intrauterine growth restriction, and preeclampsia are prevalent during yak pregnancy. Pregnancy-related diseases severely affect yak reproductive performance, causing severe losses to the yak industry and local economies [2]. The placenta plays a critical role in material exchange, hormone secretion, immune regulation, and blood flow modulation during pregnancy [3,4]; the coordinated functioning of these processes is crucial for fetal development, successful pregnancy maintenance, and pregnancy-related disease avoidance [4,5]. Therefore, investigating the structure and function of yak placenta, particularly the molecular regulatory mechanisms involved in different gestational stages, is essential for a comprehensive understanding of yak reproductive physiology and for preventing and treating pregnancy-related diseases in yaks.

Trophoblast cells (TCs), a major cell population in the placenta, help develop a stable placental structure through coordinated invasion, differentiation, and secretory functions, providing critical support for fetal growth and development [6]. Defects in TC adhesion and junction formation lead to structural abnormalities in the placenta and contribute to various pregnancy-related diseases [7]. For example, preeclampsia, a hypertensive pregnancy disorder caused by reduced TC invasion and impaired vascular remodeling, often leads to postpartum hemorrhage, intrauterine fetal growth restriction, or even fetal death [8]; intrauterine growth restriction increases the risk of fetal hypoxia, acidosis, and hypoglycemia [9]; and placental abruption, typically resulting from weak TC adhesion to the uterine wall, can lead to maternal renal failure, uterine bleeding, and fetal asphyxia [10]. Recurrent miscarriages are also a common consequence of impaired TC adhesion and junction formation [7,11]. Research on TC adhesion and junctional defects in pregnancy-related disorders is well established in humans but remains limited in yaks. Thus, exploring the molecular mechanisms underlying TC adhesion and junction formation is crucial for understanding normal placental development and its role in pregnancy in yaks.

Intercellular adhesion and junction formation, which occur through interactions between cell adhesion molecules (e.g., integrins and cadherins) and the extracellular matrix (ECM), regulate cell migration, differentiation, and proliferation [12]. Disruption of these adhesive interactions leads to reduced cell migration, limited differentiation, and an unstable tissue structure [13,14], which is also common in TCs and can impede placental growth and destabilize the maternal–fetal interface. In humans and cattle, several signaling pathways regulate TC adhesion and junction formation, for example, the integrin/focal adhesion kinase signaling pathway promotes TC migration and differentiation by modulating interactions between the cytoskeleton and ECM [13]; the mitogen-activated protein kinase signaling pathway, activated by external growth factors such as transforming growth factor beta and epidermal growth factor, enhances TC proliferation during mid-pregnancy, maintaining the stability of the maternal–fetal interface and ensuring fetal development [15]; and the wingless/integrated (Wnt) signaling pathway plays a vital role in TC adhesion and junction formation, helping maintain placental function and structural stability [16,17]. In summary, TC adhesion and junction formation are indispensable for normal placental development and pregnancy progression. Despite these advances in other species, little is known about the molecular regulation of TC adhesion and junction formation in yaks, particularly across different gestational stages. Accordingly, the aim of this study was to explore the dynamic differentiation process and potential molecular mechanisms of TCs in yak placental tissues at two and five months of gestation. Differentially expressed proteins (DEPs) associated with cell adhesion and junction formation were identified using data-independent acquisition (DIA) proteomics and bioinformatics analysis. The functional roles and expression patterns of key DEPs were systematically verified using morphological assessments and molecular biology techniques. This research provides a novel and comprehensive understanding of TC adhesion and junction formation during yak pregnancy, contributing to improved strategies for preventing pregnancy-related disorders and enhancing yak reproductive performance.

## 2. Materials and Methods

### 2.1. Sample Preparation and Collection

Samples were obtained from a commercial slaughterhouse in Tianzhu Tibetan Autonomous County (Wuwei City, Gansu Province, China). Healthy pregnant yaks (six years old) with a normal metabolism and no evidence of disease were selected. The pregnancy stage of the fetuses was determined and classified based on the crown–rump length [18] and information provided by the herders. Uterine tissues were immediately collected after slaughter and rinsed three to five times with physiological saline. The uteri were then dissected using sterile surgical scissors to expose the fetal membranes, ensuring the integrity of the amnion, allantois, and chorion. Placental tissues corresponding to early pregnancy (two-month gestation group) and middle pregnancy (five-month gestation group) stages were collected (*n* = 3/group). A portion of the tissues was fixed in 4% paraformaldehyde, and the remaining tissues were stored at −80 °C for subsequent analyses. This study was approved by the Ethics Committee of Gansu Agricultural University (code GSAU-Eth-LST-2021-003).

### 2.2. Hematoxylin–Eosin (H&E) Staining

Fixed placental tissues were processed following standard paraffin-embedding protocols and sectioned into 5 μm thick slices using a microtome (Leica, Wetzlar, Germany) [19]. Sections were deparaffinized in xylene and rehydrated using a graded ethanol series. H&E staining was performed as described previously [19,20]. Briefly, nuclei were stained with hematoxylin, followed by differentiation and bluing treatment. The cytoplasm was then counterstained with eosin, followed by gradient dehydration. Finally, the sections were sealed with neutral balsam (Servicebio, Wuhan, China). All images were captured using an optical microscope and image processing system (Nikon, Tokyo, Japan). All staining assays were performed at least in triplicate.

### 2.3. Immunofluorescence (IF) Staining

IF staining assays were performed for the co-localization analysis of occludin, intercellular adhesion molecule-1 (ICAM1), β-catenin (CTNNB1), E-cadherin (CDH1), and cytokeratin (CK)-7 proteins, as previously described [20,21]. Specifically, the sections were incubated with sodium citrate for antigen retrieval and with H_2_O_2_ to inactivate endogenous peroxidase. After blocking, the sections were incubated with primary antibodies at different dilutions (Table S1), then incubated with appropriate secondary antibodies (Alexa Fluor^®^ 488 for CK-7, Alexa Fluor^®^ 647 for CTNNB1 and occludin, and Alexa Fluor^®^ 594 for CDH1 and ICAM1; Abcam, Cambridge, UK), as described previously [20,21,22]. Nuclei were stained with 4′,6-diamidino-2-phenylindole (DAPI) solution (Solarbio, Beijing, China). Fluorescence signals and images were captured using a fluorescence microscope (ECHO; Broomfield, CO, USA). All staining assays were performed at least in triplicate.

### 2.4. DIA Sequence and Bioinformatics Analysis

Raw DIA proteomics data were acquired using an Orbitrap Exploris 480 system (Thermo Fisher Scientific, Waltham, MA, USA) coupled with nano-LC, where peptides are separated on a C18 column and analyzed using the DIA method with multiple isolation windows covering a broad *m*/*z* range, as previously described [23]. The acquired spectra were then processed using SpectronAut v19.7 (Biognosys AG, Schlieren, Switzerland) to identify and quantify peptides based on a reference protein database with raw DIA data mapped to the reference genome (NCBI-GCA_005887515.3) and analyzed using default parameters to obtain the protein quantification results. A 1% *Q* value (false discovery rate) cutoff was applied at both the precursor and protein levels. Proteins were quantified using the top three peptides that passed the 1% *Q* value cutoff. DEPs were identified based on an absolute fold change > 1.50 and a *p* value < 0.05 for functional annotation and analysis. Gene Ontology (GO) and Kyoto Encyclopedia of Genes and Genomes (KEGG) pathway analyses were conducted to annotate the functions of DEPs, as described previously [20], with terms and pathways considered significant at *p* < 0.05. DIA proteomic sequence data from the placental tissues of two-month and five-month gestation groups of yaks, with accession numbers IPX0006098000/PXD040910 in the ProteomeXchange database (https://www.iprox.cn/page/project.html, accessed on 12 July 2022), were used for GO and KEGG annotation and analysis. We focused on DEPs and GO terms (*p* < 0.01) related to cell adhesion and junction formation. Enrichment analysis of key DEPs was performed using the KEGG pathway database (*p* < 0.05) to further elucidate DEP functions. Enrichment circle plots, volcano plots, heatmaps, and UpSet–Venn diagrams were created using the OmicShare online platform (http://www.omicshare.com/tools, accessed on 20 November 2024) [19,21]. Protein–protein interaction networks of candidate DEPs were generated using STRING v12.0 (EMBL, Heidelberg, Germany) and Cytoscape 2.8.1 (Cytoscape Consortium, La Jolla, CA, USA) using the ClueGO plugin (Cytoscape Consortium, La Jolla, CA, USA) [24,25]. The potential mechanisms of the candidate DEPs were inferred through KEGG analysis and a literature review. Illustrative mechanistic diagrams were generated using Adobe Illustrator 2022 (San Jose, CA, USA).

### 2.5. Immunohistochemistry (IHC) Staining

After deparaffinization in xylene and rehydration in graded ethanol, sections were treated with sodium citrate (Beyotime, Shanghai, China) for antigen retrieval. Subsequently, the sections were incubated sequentially with 3% H_2_O_2_ and bovine serum albumin (Solarbio, Beijing, China) to block non-specific binding, followed by overnight incubation at 4 °C with the primary antibodies at different dilutions. IHC staining was performed using a Streptavidin-Biotin Complex (Bioss, Beijing, China) staining system according to the manufacturer’s instructions [20,22]. Positive signals were visualized using a 3,3′-diaminobenzidine detection kit (Service Bio, Wuhan, China). All images were captured and analyzed using an optical microscope and image processing system (Nikon, Tokyo, Japan). All staining assays were performed at least in triplicate.

### 2.6. RNA Isolation, cDNA Synthesis, and Quantitative PCR Assays

Total RNA was extracted from yak placental tissues in the two groups using a FastP.RNA isolation kit (Vazyme, Nanjing, China) then used for cDNA synthesis according to the manufacturer’s instructions [19,20]. The RNA concentration was quantified using a NanoDrop-8000 (Thermo Fisher Scientific, Waltham, MA, USA), with RNA integrity evaluated using 1% denaturing formaldehyde agarose gel electrophoresis. Subsequently, 1 μg of total RNA was reverse-transcribed into cDNA using an Evo M-MLV RT Kit (AG, Wuhan, China). The relative expression levels of *CTNNB1* and *CDH1* mRNA in the placental tissues were quantified using quantitative PCR (qPCR), as described previously [19,21]. The expression of *β-actin* was used as an endogenous control. qPCR primers (Table S2) were designed using Primer 3.0 (https://primer3.ut.ee/, accessed on 10 August 2024) and synthesized by Qingke Biotech (Xi’an, China). The qPCR reactions were performed on a LightCycler 96 real-time PCR system (Roche, Basel, Switzerland), and relative expression levels were calculated using the 2^−ΔΔCT^ method [26]. All reactions were performed in triplicate to ensure reproducibility.

### 2.7. Western Blotting

The relative expression patterns of CTNNB1 and CDH1 proteins in placental tissues at different pregnancy stages were examined by Western blot. Total protein was extracted from 100 mg of each tissue sample using pre-chilled radioimmunoprecipitation assay buffer (Solarbio, Beijing, China) with 1 mM phenylmethylsulfonyl fluoride (Solarbio, Beijing, China). Protein concentrations were measured using a bicinchoninic acid protein assay kit (Boster, Wuhan, China). A total of 30 μg protein was used for Western blot analysis, as previously described [21,27]. The proteins were then transferred to polyvinylidene fluoride membranes for co-incubation with the primary antibodies overnight at 4 °C. The membranes were then incubated with the secondary antibody, visualized using enhanced chemiluminescent reagent (New Cell & Molecular Biotech, Jiangsu, China), and imaged with a chemiluminescence imaging system (General Electric, Boston, MA, USA). The integrated optical density (IOD) values of the bands were visualized and quantified using ImageJ software v1.44p (National Institutes of Health, Bethesda, MD, USA). β-actin was used as an endogenous control, and the protein expression levels of CDH1 or CTNNB1 in the two-month gestation group were used as the reference for normalization. All immunoblot assays and statistical analyses were performed in triplicate.

### 2.8. Statistical Analysis

All data were presented as the mean ± SD. qPCR and Western blot data were analyzed using Student’s *t*-test (between two groups) or one-way analysis of variance (within multiple groups). Statistical analyses and graph construction were performed using Prism software (version 9.0; GraphPad, San Diego, CA, USA) and Adobe Illustrator (Adobe, San Jose, CA, USA). Statistical significance was set to *p* < 0.05.

## 3. Results

### 3.1. Morphology of TCs in Yak Placenta at Different Gestation Stages

**Figure 1:**
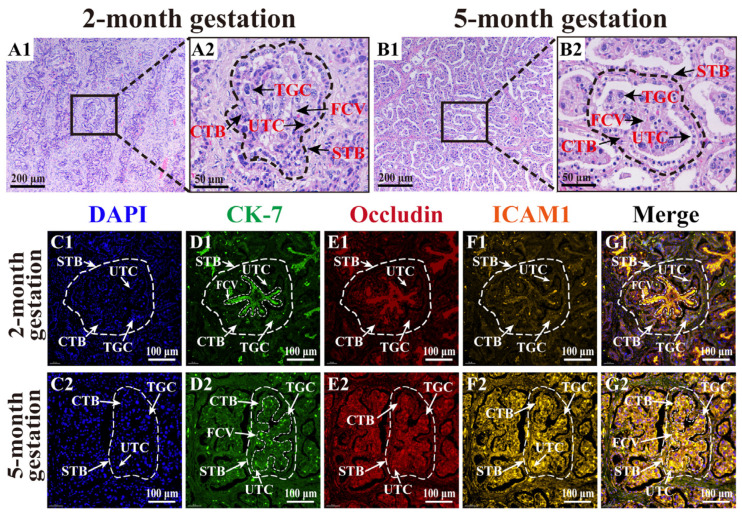
Morphological observation of yak placental tissues at two and five months of gestation. (**A**,**B**) H&E staining results. (**C**–**G**) Co-localization analysis of occludin and ICAM1: nuclei (blue, (**C1**,**C2**)), CK-7 (green, (**D1**,**D2**)), occludin (red, (**E1**,**E2**)), ICAM1 (orange, (**F1**,**F2**)), and merged CK-7, occludin, and ICAM1 staining (**G1**,**G2**). CTB, cytotrophoblast; STB, syncytiotrophoblast; UTC, uninucleate trophoblast cell; TGC, trophoblast giant cell; FCV, fetal cotyledonary villi. Scale bars: 200 μm (100× magnification), 100 μm (200× magnification), 50 μm (400× magnification).

H&E staining results at two months of gestation revealed that the chorion extended villi while forming folds, with primary villi progressively branching into shorter secondary villi with sparse blood vessels in the villous stroma (Figure 1(A1)). At this stage, the trophoblast was structured into inner cytotrophoblast (CTB) and outer syncytiotrophoblast (STB) layers, with abundant trophoblast giant cells (TGCs) and uninucleate trophoblast cells (UTCs) distributed throughout the tissue (Figure 1(A2)). At five months of gestation, the placental STB and CTB showed clear demarcation and vacuolated structures. The volume of the fetal cotyledonary villus (FCV) was significantly increased through branching into secondary/tertiary villi, enhancing the maternal–fetal interface, and a dense capillary network was observed in the villous stroma (Figure 1(B1)). The nuclei of UTCs appeared round or oval upon light staining, whereas those of multinucleated TGCs were larger and more intensely stained (Figure 1(B2)). The IF results revealed clearly defined DAPI-labeled nuclei in the placental tissues of both groups (Figure 1C). Compared with the two-month gestation group, the five-month gestation group exhibited significantly increased fluorescence intensity of the TC marker CK-7, tight junction protein occludin, and adhesion molecule marker ICAM1 (Figure 1D–F). The co-localization results showed that CK-7, occludin, and ICAM1 were widely expressed in FCV and co-localized in the cytoplasm of both TGCs and UTCs (Figure 1G). These findings highlight the dynamic structural changes that occur in the placenta during early and middle pregnancy, particularly during TC invasion and differentiation. The upregulation of occludin and ICAM1 underscores the critical role of cell adhesion and junction formation in TC invasion and differentiation.

### 3.2. Proteome Analysis and Protein Identification and Annotation Results

**Figure 2:**
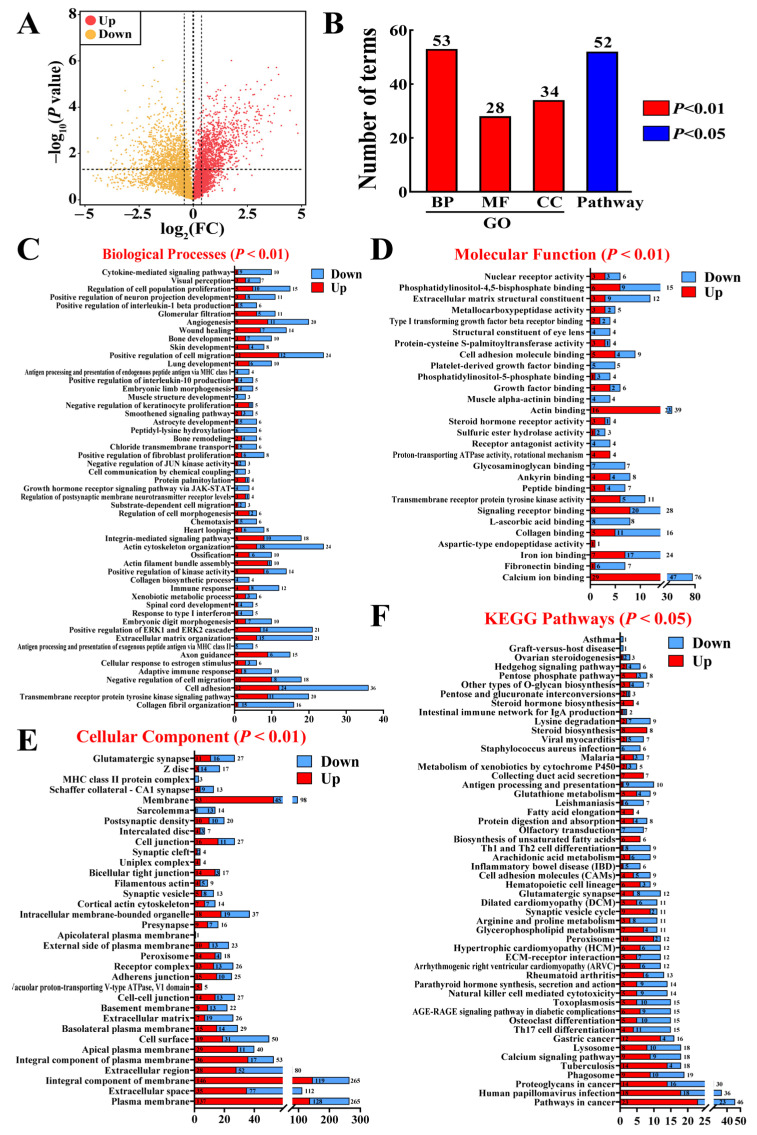
Summary of proteome analysis and protein identification and annotation results from DIA analysis. (**A**) Volcano plot of 1622 DEPs, including 836 upregulated and 786 downregulated DEPs. (**B**) Summary of GO terms for biological processes (BP), molecular functions (MF), cellular components (CC) (*p* < 0.01), and pathway annotation (*p* < 0.05). (**C**–**F**) GO annotation results for BPs, MFs, and CCs, along with pathway annotation.

Compared with the two-month gestation group, 1622 DEPs, including 836 upregulated and 786 downregulated DEPs, were screened in the five-month gestation group (Figure 2A, Table S3). These DEPs were subjected to GO term (*p* < 0.01, Table S4) and KEGG pathway (*p* < 0.05, Table S5) annotation, which identified 53 biological processes (BP), 28 molecular functions (MF), 34 cellular components (CC), and 52 pathways (Figure 2B). Most BPs were associated with cell adhesion and migration, immune responses, and developmental processes (Figure 2C). Most MFs were related to their binding activities (ions, proteins, and lipids), receptor activities, and enzymatic functions (Figure 2D). Most CCs were associated with membrane structure and function, extracellular and adhesion processes, and cell junctions (Figure 2E). KEGG enrichment analysis revealed that most pathways were related to the immune response and inflammation, cancer and cell growth, and hormone/steroid regulation and metabolism (Figure 2F). Proteome analysis identified 1622 DEPs enriched in GO terms and pathways, suggesting their relevance to the molecular mechanisms underlying placental development.

### 3.3. Identification of Candidate DEPs Related to Cell Adhesion and Junctions from GO Terms

**Figure 3:**
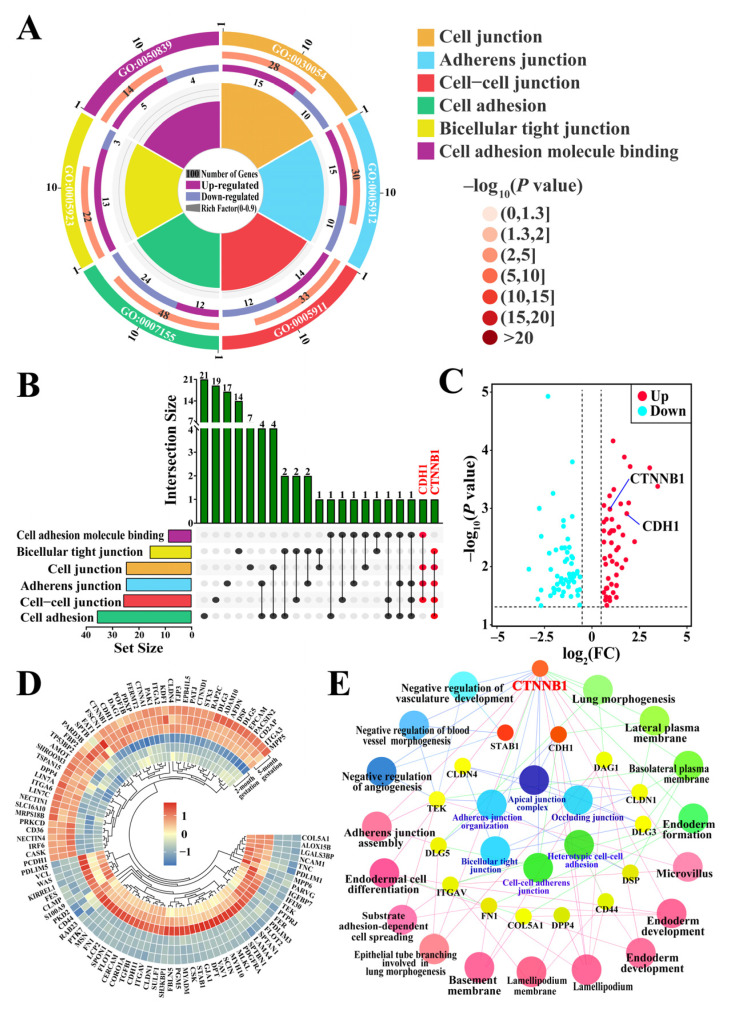
Identification of candidate DEPs related to cell adhesion and junction formation based on GO enrichment data. (**A**) Candidate DEPs and GO terms. (**B**) UpSet–Venn diagram of six GO terms and 103 DEPs. (**C**) Volcano plot of 103 DEPs, including 54 downregulated and 49 upregulated DEPs. (**D**) Heatmap of 103 candidate DEPs. (**E**) Interactive relationship analysis of candidate DEPs and GO terms performed using ClueGO.

GO terms related to cell adhesion and junction formation, along with associated candidate DEPs, were screened based on the 1622 DEPs identified from DIA proteomics (Figure 3). Six GO terms (*p* < 0.01), including cell junctions, adherens junctions, cell–cell junctions, cell adhesion, bicellular tight junctions, and cell adhesion molecule binding, and 103 candidate DEPs were identified (Figure 3A, Table S6). The UpSet–Venn diagram indicated that CTNNB1 was shared among five GO terms, whereas CDH1 was shared among four GO terms (Figure 3B). The volcano plot and heatmap further showed that the candidate proteins, which included 54 downregulated and 49 upregulated proteins, were differentially expressed between the two-month and five-month gestation groups. Notably, CTNNB1 and CDH1 were upregulated in the placental tissue at five months of gestation (Figure 3C,D). ClueGO analysis revealed that 15 of the 103 DEPs were directly related to 23 GO terms, and that the important candidate DEPs CTNNB1 and CDH1 were associated with 18 GO terms related to cell adhesion and seven GO terms related to cell junctions, respectively (Figure 3E). These findings indicate that CTNNB1 and CDH1 play pivotal roles in yak pregnancy, likely by regulating cell adhesion and junction formation.

### 3.4. Identification of Pathways and Candidate DEPs Related to CTNNB1 and CDH1

**Figure 4:**
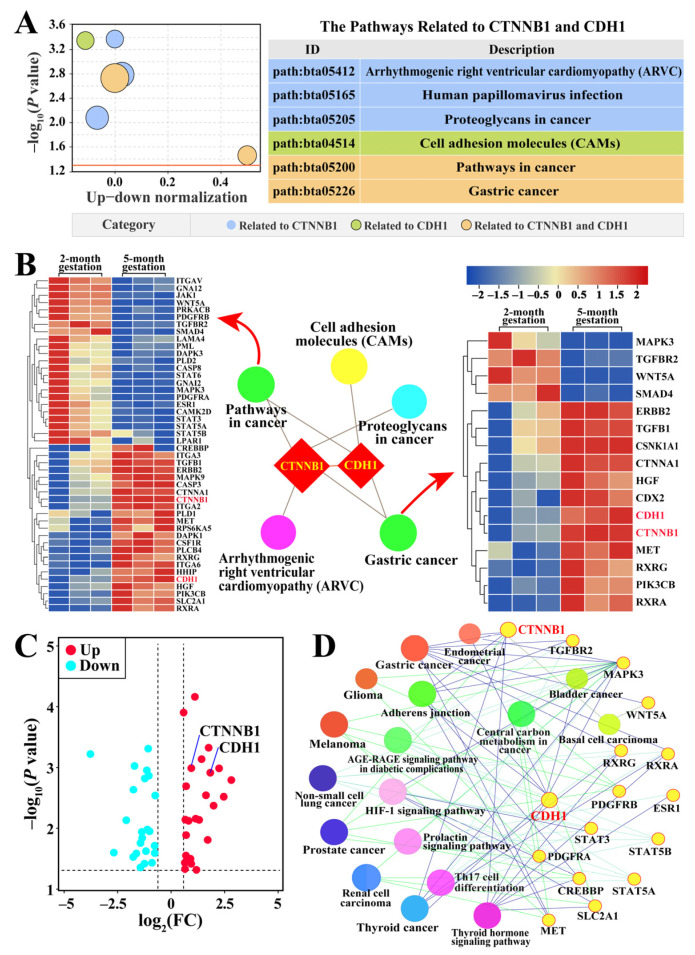
Identification of candidate DEPs and pathways related to cell adhesion and junction formation involving CTNNB1/CDH1. (**A**) Candidate DEPs and pathways. (**B**) Protein–protein interaction network and heatmap analysis of pathways. (**C**) Volcano plot of 48 DEPs, including 23 downregulated and 25 upregulated DEPs. (**D**) Interactive relationship analysis of candidate DEPs and pathways performed using ClueGO.

Pathways involving CTNNB1 and CDH1 were selected to identify their potential roles in cell adhesion and junction formation in yak placental tissues at two and five months of gestation (Figure 4). Six pathways, including cell adhesion molecules, arrhythmogenic right ventricular cardiomyopathy, proteoglycans in cancer, pathways in cancer, human papillomavirus infection, and gastric cancer, were identified by DIA proteomic analysis. Among these, gastric cancer and pathways in cancer were the two key pathways associated with CTNNB1 and CDH1 (Figure 4A, Table S7). The interaction network of these two pathways suggests that CTNNB1 is involved in four pathways, whereas CDH1 is involved in three. The DEPs associated with the two key pathways were significantly differentially expressed between the two-month and five-month gestation groups (Figure 4B). A total of 48 candidate DEPs, including 23 downregulated and 25 upregulated DEPs, were identified within these two pathways (Figure 4C). ClueGO analysis indicated that 16 of the 48 DEPs were directly related to 17 pathways, with CTNNB1 and CDH1 involved in eight pathways related to cell adhesion and junction formation and seven cancer-related KEGG pathways, respectively (Figure 4D). These findings suggest that CTNNB1 and CDH1 may regulate placental development during pregnancy, likely through their involvement in cell adhesion and junction formation, as well as cancer-related pathways.

### 3.5. Distribution and Co-Localization Analysis of CTNNB1 and CDH1 Proteins in Yak Placental Tissues

**Figure 5:**
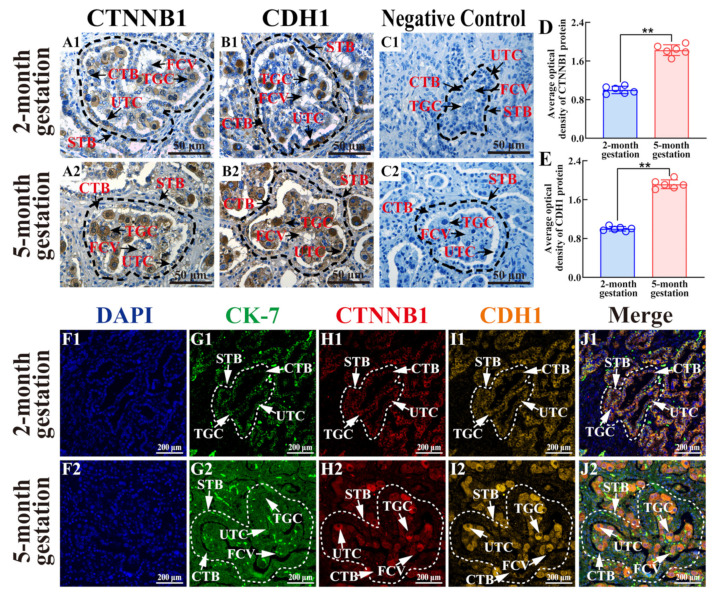
Distribution and co-localization analysis of CTNNB1/CDH1 in yak placental tissues at two and five months of gestation. (**A**,**B**) Intracellular localization analysis. (**C**) Negative control. (**D**,**E**) Gray values of positive expression of CTNNB1 and CDH1 proteins quantified using ImageJ software. (**F**–**J**) Co-localization analysis: nuclei (blue, (**F1**,**F2**)), CK-7 (green, (**G1**,**G2**)), CTNNB1 (red, (**H1**,**H2**)), CDH1 (orange, (**I1**,**I2**)), merged with CK7, CTNNB1, and CDH1 (**J1**,**J2**) staining. CTB, cytotrophoblast; STB, syncytiotrophoblast; UTC, uninucleate trophoblast cell; TGC, trophoblast giant cell; FCV, fetal cotyledonary villi. Scale bars: 200 μm (100× magnification), 50 μm (400× magnification). ** represents *p* < 0.01.

Positive expression of CTNNB1 and CDH1 was observed in the FCV and cytoplasm of TCs at both two and five months of gestation (Figure 5A,B). In the two-month gestation group, CTNNB1 and CDH1 were detected in UTCs and TGCs and sparsely detected in FCV (Figure 5(A1,B1)). The staining intensity of CTNNB1 and CDH1 was enhanced at five months of gestation in UTCs, TGCs, and FCV (Figure 5(A2,B2)). No positive staining for CTNNB1 or CDH1 was observed in the negative control group (Figure 5(C1,C2)). IOD analysis revealed significantly upregulated expression of CTNNB1 and CDH1 in the five-month gestation group compared to that in the two-month gestation group (*p* < 0.01) (Figure 5D,E). The IF results revealed clearly defined nuclei labeled with DAPI in placental tissues at both two and five months of gestation (Figure 5F). The fluorescence intensity of CK-7 was significantly higher at five months of gestation than at two months of gestation (Figure 5G). Positive fluorescence signals for CTNNB1 and CDH1 were present in the cytoplasm of TGCs and UTCs in both two-month and five-month gestation groups, with stronger signal intensity in the latter group (Figure 5H,I). The co-localization results indicated that CK-7, CTNNB1, and CDH1 were co-localized in the cytoplasm of TGCs and UTCs (Figure 5J). These findings highlight the upregulated expression of CTNNB1 and CDH1 and their cooperative roles in TC adhesion and junction formation during pregnancy.

### 3.6. CTNNB1 and CDH1 mRNA and Protein Expression Patterns in Yak Placental Tissues

**Figure 6:**
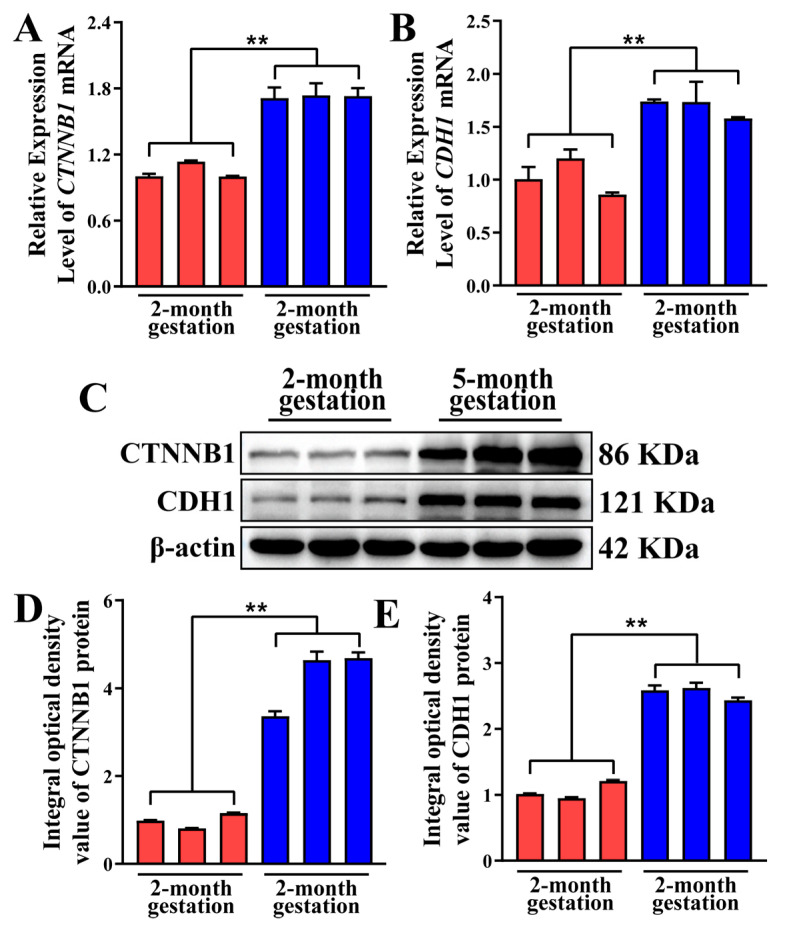
Pattern analysis of *CTNNB1* and *CDH1* mRNA and protein expression in yak placental tissues. (**A**,**B**) Expression levels of *CTNNB1* and *CDH1* mRNA monitored by qPCR. (**C**) Protein bands of CTNNB1, CDH1, and β-actin in placental tissues. (**D**,**E**) Integral optical density values of CTNNB1 and CDH1 proteins in yak placental tissues, respectively. Data are presented as means ± SEM. ** represents *p* < 0.01.

To further investigate the impact of placental TC adhesion and junction formation on pregnancy, the relative expression levels of *CTNNB1* and *CDH1* mRNA and protein were evaluated in yak placental tissues using qPCR and Western blot (Figure 6). Compared to the two-month gestation group, the five-month gestation group showed significantly upregulated relative mRNA expression levels of *CTNNB1* and *CDH1* (*p* < 0.01) (Figure 6A,B). Moreover, CTNNB1 and CDH1 protein expressions were present but were different in each sample of the two groups (Figure 6C). IOD values indicated that CTNNB1 and CDH1 expressions were significantly higher in the five-month gestation group than in the two-month gestation group (*p* < 0.01) (Figure 6D,E). Overall, these findings suggest that CTNNB1 and CDH1 may play critical roles in maintaining placental structure and function, and that their upregulation is strongly correlated with TC adhesion and junctional integrity in the placenta.

## 4. Discussion

Cell adhesion and junction formation are critical to maintaining TC structural stability and functional integrity, which directly influence placental development and pregnancy maintenance [12,28]. Abnormalities in these processes can disrupt placental growth, material transport, immune regulation, maternal–fetal interface stability, and placental tissue remodeling [7,11]. Thus, investigating TC morphological changes and identifying key functional proteins involved in cell adhesion and junction formation can offer valuable insights into the mechanisms underlying yak placental development.

Morphological changes in TCs during early- and mid-pregnancy are essential for normal placental development [29,30]. During early pregnancy, TCs migrate and invade the surrounding decidua and maternal blood vessels, forming an intervillous space serving as the foundation for placental growth and function [31]. In middle pregnancy, TC invasiveness decreases, whereas adhesion, junction formation, and differentiation capacities increase, leading to clearer boundaries between STBs and CTBs, thus ensuring maternal–fetal nutrient exchange and pregnancy stability [32]. Likewise, our findings suggest that TC invasion, migration, and differentiation are closely associated with cell adhesion and junction formation. TC invasiveness and migration are finely regulated by the uterine microenvironment, with various proteases and adhesion molecules being directly or indirectly involved in the infiltration and adhesion processes [8,12]. For example, increased ICAM1 expression in TCs promotes the adhesion between trophoblasts and endometrial cells, facilitating proper differentiation and syncytiotrophoblast formation [33]. Additionally, occludin, a key tight junction protein, is positively expressed in both STBs and CTBs, sealing intercellular gaps and preventing the non-selective permeability of ions, water, and other molecules, thus maintaining placental barrier function and preventing immune rejection [12,34]. Compared to early pregnancy TCs, those in mid-pregnancy showed significantly upregulated expression of occludin and ICAM1, highlighting the importance of cell adhesion and junction formation processes in placental homeostasis. This phenomenon may result from the increasing demand for enhanced placental barrier function as pregnancy progresses, which increases cell junction protein expression. During mid-pregnancy, the increased need for maternal–fetal material exchange promotes stable cell adhesion and tight junction formation, maintaining placental structure and function [34,35]. However, the relationship between these processes and placental development, and the underlying molecular mechanisms, requires further investigation in yaks.

Based on DIA proteomics data, we identified six key GO terms and 103 DEPs, particularly CTNNB1 and CDH1, involved in cell junctions, adhesion junctions, and cell adhesion GO terms and are likely critical to regulating placental development in yaks. CTNNB1 and CDH1 form cell adhesion complexes that promote stable intercellular adhesion and cell polarity [35,36]. This adhesion mechanism is essential for placental barrier function. Specifically, CDH1 expression level regulates trophoblast cell invasiveness, with lower expression in early pregnancy facilitating trophoblast migration, while upregulation in mid-pregnancy contributes to establishing and stabilizing the placental barrier [37,38]. CTNNB1 regulates cell proliferation and differentiation through the Wnt signaling pathway, further influencing placental maturation [17,35,39]. Additionally, CTNNB1 and CDH1 are involved in endoderm formation during early embryonic development; however, their precise mechanisms require further exploration [40]. Pathway analysis revealed that they participate in cancer-related pathways, including gastric cancer and pathways in cancer. CTNNB1 regulates cell proliferation and differentiation through the Wnt signaling pathway, and its mutations can lead to uncontrolled cell proliferation, suppressed apoptosis, and activation of tumor invasion and metastasis [35,36]. Downregulation of CDH1 disrupts intercellular adhesion, promotes epithelial–mesenchymal transition in tumor cells, and enhances their invasive and metastatic capabilities [41]. Similarly to the regulation of cancer cell proliferation, TC adhesion and junction functions during placental development may be regulated by CTNNB1 and CDH1. Abnormal expression of CTNNB1 and CDH1 was linked to pregnancy-related placental disorders. For example, in patients with preeclamptic, dysfunction and abnormal differentiation of the CTB in the placenta are linked to the dysregulated expression of CTNNB1 and CDH1 [42]. Additionally, gestational diabetes mellitus may be linked to excessive CTNNB1 activation, which interferes with placental metabolic function and downregulates CDH1, thus disrupting placental barrier function [42]. ClueGO analysis suggested that other potential key DEPs in related GO processes and cancer-related pathways may also regulate placental development and pregnancy processes, warranting further investigation. For example, cluster of differentiation 44 [43] in endoderm development, stabilin-1 [44] in the negative regulation of vasculature development, and fibronectin [45] and integrin subunit alpha V [46] in substrate adhesion-dependent cell spreading promote TC migration and invasion, maintain placental stability, and facilitate embryo implantation. Importantly, interactions between CTNNB1/CDH1 and these key DEPs highlight their critical role in placental development.

CTNNB1 and CDH1 form an adhesion complex that maintains tight intercellular connections during cell adhesion [35,36]. Mutations in CTNNB1/CDH1 may disrupt Wnt signaling, hinder TC adhesion during embryonic development, impair embryo implantation, and affect normal fetal development [17,39,40]. CTNNB1/CDH1 are critical to inhibiting cell migration and maintaining normal embryonic development during pregnancy, suggesting that they may regulate pregnancy through cell adhesion and junction processes. Our results are consistent with such findings, but studies on the role of CTNNB1/CDH1 in yak placental development during pregnancy are limited. Our DIA proteomics results showed that CTNNB1/CDH1 expression was higher in mid-pregnancy, suggesting its involvement in TC adhesion and junction formation in yak placental tissues, which is consistent with the mRNA and protein expression results. CTNNB1, a cytoskeletal component, forms complexes with CDH1 on the inner side of the epithelial cell membrane, promoting cell adhesion, morphological stability, and signal transduction via cytoskeletal interactions [47,48]. CTNNB1 is localized in the cytoplasm and involved in cytoskeletal connections [48], whereas CDH1 is localized in the Golgi apparatus and participates in intracellular transport and degradation processes [49]. CTNNB1/CDH1 is primarily localized in the cell membrane and cytoplasm, maintaining intercellular adhesion and tissue structure integrity [47,48,49]. We confirmed that CTNNB1/CDH1 was predominantly expressed in the cytoplasm of FCV, UTCs, and TGCs, suggesting that high CTNNB1/CDH1 expression in mid-pregnancy supports TC adhesion, junction formation, and differentiation, thereby contributing to the gradual development and stability of the placental structure and ensuring pregnancy progression.

**Figure 7:**
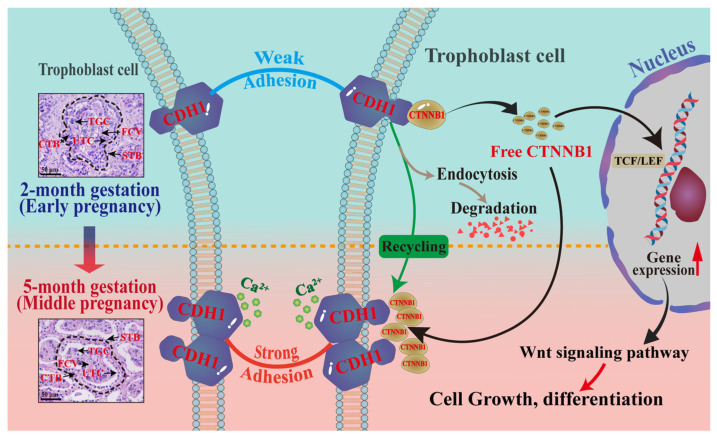
Potential molecular mechanisms by which CTNNB1 and CDH1 mediate adhesion and junction formation in placental trophoblast cells. Scale bar: 50 μm (400× magnification).

Based on these results and those of previous studies, we propose a potential mechanism underlying the roles of CTNNB1 and CDH1 in pregnancy (Figure 7). During pregnancy, TCs in the placenta undergo key adhesion changes, transitioning from weak to strong intercellular adhesions to appropriate invasion and subsequent stabilization, which are essential for placental development [12,34]. Specifically, early in pregnancy, limited CDH1 expression results in weaker adhesion between TCs, promoting their migration into and invasion of the uterine lining [12,50]. CTNNB1 was internalized and degraded, further reducing adhesion strength [51]. As pregnancy progresses to mid-pregnancy, the increased CDH1 and CTNNB1 expression enhance adhesion by forming stable CTNNB1–CDH1 complexes aided by calcium ions (Ca^2+^) [37]. Additionally, recycling of CTNNB1 to the cell membrane further strengthens adhesion, providing stability to the developing placenta [38]. Free CTNNB1 can also activate the Wnt signaling pathway, promoting TC growth and differentiation [39]. Therefore, the dynamic regulation of CTNNB1/CDH1 is crucial for maintaining the balance between TC invasion and stable adhesion during placental development.

However, our study has limitations. Specifically, further validation using TC experiments and pregnancy animal models is required to verify the mechanisms of cell adhesion and junction formation and improve the reliability of our findings. Nevertheless, we have systematically identified candidate DEPs associated with cell adhesion and junction formation, providing new insights into the molecular mechanisms underlying yak placental development during pregnancy.

## 5. Conclusions

This study revealed the crucial role of CTNNB1/CDH1 in maintaining placental homeostasis and regulating trophoblast adhesion and junctions in yaks during pregnancy. Based on DIA proteomics data, we identified 103 DEPs associated with six GO terms and 48 DEPs associated with two pathways. Bioinformatics analysis indicated that CTNNB1 and CDH1 are involved in placental development and pregnancy maintenance via cell adhesion and junction processes as well as cancer-related pathways. Furthermore, IHC and IF staining localized CTNNB1 and CDH1 predominantly in the cytoplasm of TCs, especially UTCs and TGCs. The mRNA and protein expression levels of *CTNNB1* and *CDH1* were significantly upregulated at five months of gestation compared to that at two months of gestation. In conclusion, this study provides new insights into the molecular mechanisms underlying TC adhesion and junction formation during yak placental development, as well as a theoretical foundation for improving yak reproductive performance.

## Data Availability

The data that support the findings of this study are available from the corresponding author upon reasonable request.

## Supplementary Materials

The following supporting information can be downloaded at: https://www.mdpi.com/article/10.3390/ani15060876/s1. Table S1: The used antibodies and dilution ratio in the present study. Table S2: qPCR primer sequences. Table S3: The 1622 DEPs generated from the DIA proteomics sequence database. Table S4: Data of KEGG pathway with *p* < 0.05. Table S5: Data for GO terms with *p* < 0.05. Table S6: The six significant GO terms and 103 DEPs identified from DIA proteomics. Table S7: The six significant KEGG pathways and 91 DEPs identified from DIA proteomics. Figures S1–S3: Whole Western blot of CTNNB1 (86 kDa), CDH1 (121 kDa), and β-actin (42 kDa).
