# Supplementary figures and images for: CTNNB1 and CDH1 Regulate Trophoblast Cell Adhesion and Junction Formation in Yak Placental Tissue at Different Gestational Stages
Source: Animals (Basel). 2025 Mar 19;15(6):876. doi: 10.3390/ani15060876 (PMC11939409; doi:10.3390/ani15060876)

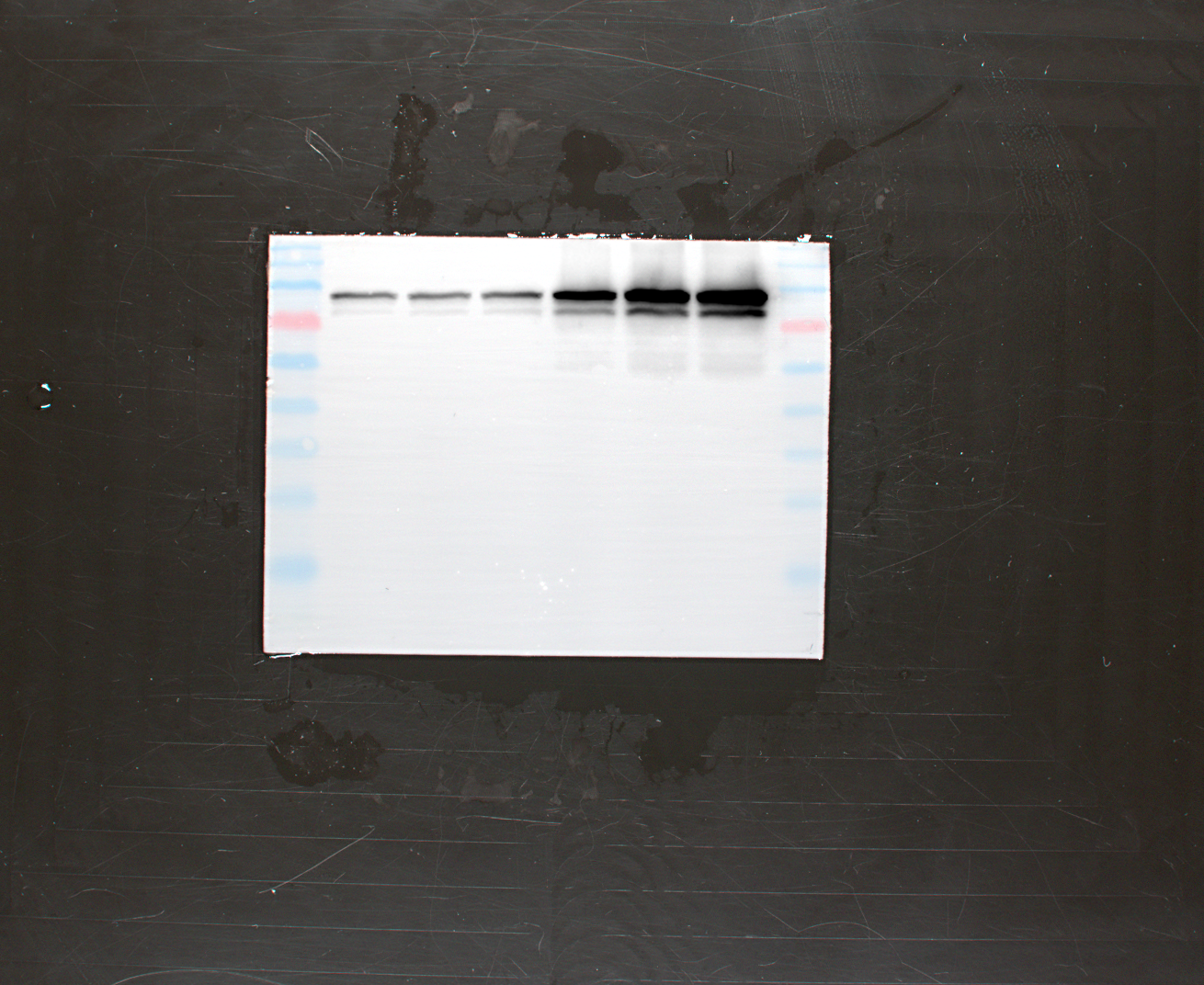

Supplement: Supplementary file 1 [file animals-15-00876-s001.zip › Figure S1 CTNNB1.Tif]

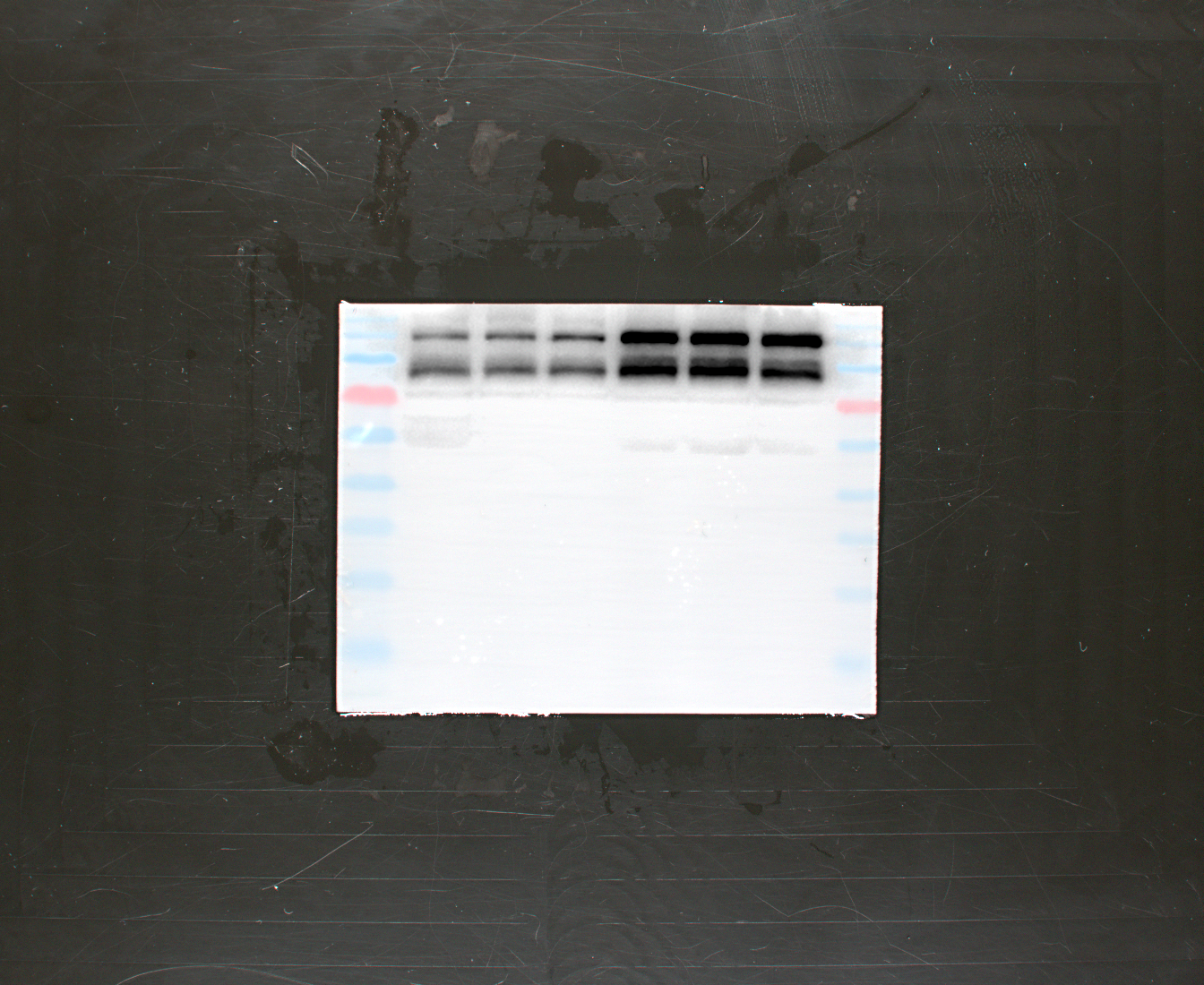

Supplement: Supplementary file 1 [file animals-15-00876-s001.zip › Figure S2 CDH1.Tif]

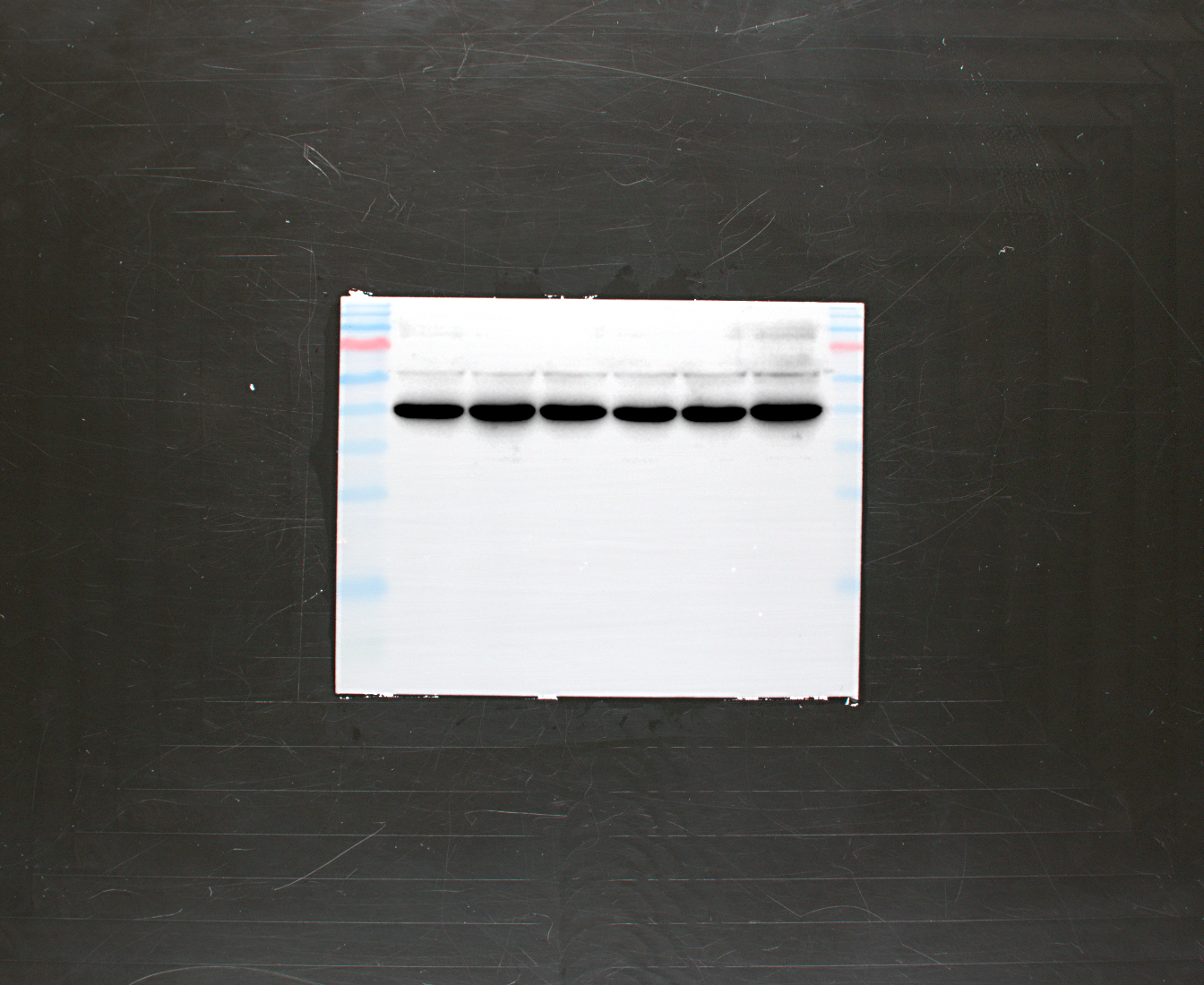

Supplement: Supplementary file 1 [file animals-15-00876-s001.zip › Figure S3 β-actin.Tif]
